# Nanoparticle-based targeted delivery of pentagalloyl glucose reverses elastase-induced abdominal aortic aneurysm and restores aorta to the healthy state in mice

**DOI:** 10.1371/journal.pone.0227165

**Published:** 2020-03-27

**Authors:** Saphala Dhital, Naren R. Vyavahare

**Affiliations:** Department of Bioengineering, Clemson University, Clemson, SC, United States of America; Max Delbruck Centrum fur Molekulare Medizin Berlin Buch, GERMANY

## Abstract

**Aim:**

Abdominal aortic aneurysms (AAA) is a life-threatening weakening and expansion of the abdominal aorta due to inflammatory cell infiltration and gradual degeneration of extracellular matrix (ECM). There are no pharmacological therapies to treat AAA. We tested the hypothesis that nanoparticle (NP) therapy that targets degraded elastin and delivers anti-inflammatory, anti-oxidative, and ECM stabilizing agent, pentagalloyl glucose (PGG) will reverse advance stage aneurysm in an elastase-induced mouse model of AAA.

**Method and results:**

Porcine pancreatic elastase (PPE) was applied periadventitially to the infrarenal aorta in mice and AAA was allowed to develop for 14 days. Nanoparticles loaded with PGG (EL-PGG-NPs) were then delivered via IV route at 14-day and 21-day (10 mg/kg of body weight). A control group of mice received no therapy. The targeting of NPs to the AAA site was confirmed with fluorescent dye marked NPs and gold NPs. Animals were sacrificed at 28-d. We found that targeted PGG therapy reversed the AAA by decreasing matrix metalloproteinases MMP-9 and MMP-2, and the infiltration of macrophages in the medial layer. The increase in diameter of the aorta was reversed to healthy controls. Moreover, PGG treatment restored degraded elastic lamina and increased the circumferential strain of aneurysmal aorta to the healthy levels.

**Conclusion:**

Our results support that site-specific delivery of PGG with targeted nanoparticles can be used to treat already developed AAA. Such therapy can reverse inflammatory markers and restore arterial homeostasis.

## Introduction

An abdominal aortic aneurysm (AAA) is the 13^th^ leading cause of death in the elderly. The common characteristic of AAA disease includes the degradation of the aortic extracellular matrix, smooth muscle cell apoptosis, and gradual weakening and dilation of the aorta [1]. AAA is diagnosed when the aortic diameter is expanded by 50% or more or exceeds 3 cm. In clinical practice, if the diameter reaches 5 cm or more, patients are recommended for surgical intervention. The contributing factors for AAA include male sex, age, genetic factors, hypertension, and smoking history [1,2].

In AAA, ECM degradation occurs because of the inflammatory process. As the inflammation progresses, activated cells secret pro-matrix metalloproteinases (MMPs). The enzymatic activity of MMPs such as MMP-2, MMP-9, and MMP-12 degrade ECM specifically elastic laminae in the medial layer. Since elastin degradation is one of the first steps during the onset of the AAA, we have been working on developing a drug delivery system that targets degraded elastin at the site of AAA disease. Previously, we have shown that such targeted delivery can deliver agents to reverse calcification of arteries and reverse aortic aneurysms in calcium-chloride (CaCl_2)_ injury rat model [3]. We have shown that polyphenols such as pentagalloyl glucose (PGG) and Epigallocatechin gallate (EGCG) can increase elastin deposition by smooth muscle cells derived from healthy or aneurysmal rat aorta [4]. Others have shown than in an elastase model of AAA, a high dose of grape seed polyphenol used orally has a protective role for elastin, and decrease immune cells and MMPs at the AAA site [5]. Green tea polyphenol EGCG was used orally to a rat model of abdominal aortic aneurysm induced by intraluminal infusion of elastase and adventitial simultaneous CaCl_2_ application where EGCG prevented the progression of AAA [5]. These studies used excessively high oral doses of polyphenols at the onset of AAA induction and showed an only protective effect. Moreover, grape seed extracts can have mixtures of multiple polyphenols and other ingredients. We have been studying the development of targeted delivery of drugs to the site of aneurysms so that a minimal dose of drug will be locally delivered in a sustained release manner to not only prevent aneurysm development but to regress developed aneurysms, which is clinically more relevant. Here, we successfully demonstrate that such targeted delivery of pentagalloyl glucose (PGG) restores degraded elastin, reduces MMP activity and infiltration of inflammatory cells, and regresses already developed aneurysms in elastase-induced AAA model.

## Materials and methods

### Preparation of DIR loaded albumin nanoparticles for *in vivo* targeting studies

DiR (1, 1-dioctadecyl-3, 3, 3, 3-tetramethylindotricarbocyanine iodide) (PromoCell GmbH, Heidelberg, Germany) loaded BSA (Seracare, Milford, MA) nanoparticles were prepared by a similar method as described previously [3,6]. Briefly, bovine serum albumin, BSA (250 mg) was dissolved in DI water (4 mL) and then DiR dye (25 mg suspended in acetone) was added to BSA solution and stirred for one hour following the addition of glutaraldehyde (EM grade 70%, EMS, PA, USA) at a concentration of 42 μg/mg BSA. The mixture was added dropwise to 24 mL of ethanol under sonication (Omni Ruptor 400 Ultrasonic Homogenizer, Omni International Inc, Kennesaw, GA) on ice for 30 minutes.

### Preparation of Pentagalloyl Glucose (PGG) loaded BSA nanoparticles

PGG-loaded nanoparticles were obtained by dissolving 250 mg of bovine serum albumin, BSA (Seracare, MA) in 4 mL of deionized (DI) water as stated previously. PGG (125 mg) was dissolved in 400 μl of dimethyl sulfoxide (DMSO) then the solution was added to BSA solution. Glutaraldehyde solution at a concentration of 12μg/mg of protein (BSA) was added while stirring. After an hour of continuous stirring, the mixture was added dropwise to 24 mL of ethanol under continuous probe sonication. The sonication was continued for an additional 30 mins. The nanoparticles were separated by centrifugation and washed.

### Conjugation of the anti-elastin antibody to nanoparticles

PGG or DiR loaded BSA NPs were PEGylated (NHS-PEG (2000) Maleimide) (Avanti Polar Lipids, Inc., Alabaster, AL) to achieve a sulfhydryl-reactive particle system. A polyclonal anti-elastin antibody (custom-made at Clemson University) was thiolated with Traut’s reagent (G-Biosciences, Saint Louis, MO) according to the manufacturer's protocol. Thiolated antibodies obtained were added to the PEGylated NPs (4 μg antibody per 1 mg NPs) and incubated overnight. [7,3] The PGG loaded and elastin antibody conjugated nanoparticles are named as EL-PGG-NPs, while DiR loaded NPs are named as EL-DiR-NPs.

To study NP targeting in vivo, we also made elastin antibody conjugated gold nanoparticles (EL-GNPs) as described previously [8]. (details of methods, size, charge of NPs are presented in the supplement S1 File, and S1 Fig).

### Elastase mediated AAA in mice

AAA was induced in specific pathogen-free C57BL/6 background male mice obtained from The Jackson Laboratory (Bar Harbor, ME). Mice were accommodated 3–5 per cage, at 22–24°C, 40–55% humidity and 12-hr light/dark-light cycle. The studies were carried out with approval from the Clemson University Institutional Animal Care and Use Committee (IACUC) following the guidelines of the Clemson University Animal Research Committee. Mice receive humane care in compliance with NIH Public Law 99–158.

Briefly, to induce AAA, the mouse was anesthetized with isoflurane, and a laparotomy exposed the abdominal aorta. Porcine pancreatic elastase (PPE; Sigma-Aldrich Co., St. Louis, MO, 7.6 mg/ml) was applied peri-adventitially for 12 mins, and subsequently, the aorta was rinsed with sterile PBS [9,10]. After the abdominal specimens were placed in the original order, the fascial layers were closed with sutures. Each mouse was transferred to a new cage and was closely monitored for 24 hrs. Progression of disease in animals was monitored via high-frequency ultrasound imaging (Vevo2100, VisualSonics, Toronto, Canada). Sham group aortas were treated with PBS (no elastase).

After the surgery, mice were kept for two weeks to allow aneurysm development. Two weeks after elastase application, when significant aneurysms were developed, animals received two tail-vein injections of EL-PGG-NPs (10 mg/kg body weight) one week apart. Freshly prepared particles were delivered in sterile PBS. Control animals did not receive any therapy (n-10 per group). All animals were sacrificed at four weeks.

**Fig 1:**
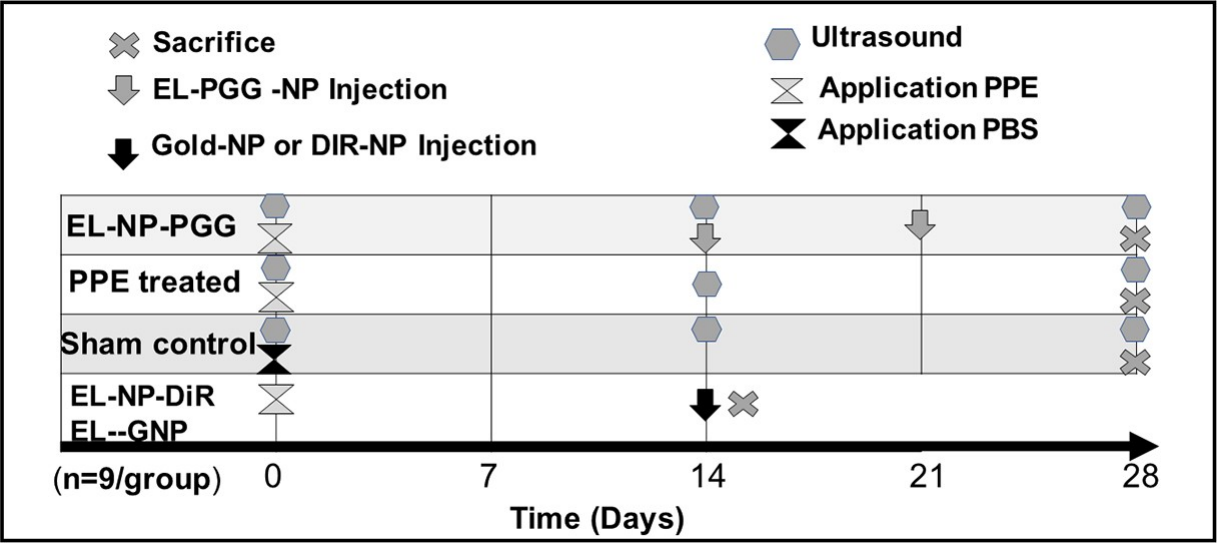
Schematic of study design.

Details of animal study design are shown in Fig 1.

### Ultrasound analysis of the aneurysms

The percentage dilation and the circumferential strain of the aorta were assessed by using the ultrasound system. The animals were anaesthetized by carefully supplying 1% to 3% isoflurane during imaging and placed on the imaging table in a supine position. Mouse heartbeat rate and body temperature were also monitored during the imaging process. Sagittal and transverse images of aortas were obtained in motion mode, B mode and color doppler mode. Systolic and diastolic inner diameters were measured and recorded at three different regions on each aneurysm or parent vessel using the built-in ultrasound software. The diastolic-to-systolic circumferential Green-LaGrange strains were analyzed using axial symmetry. The Circumferential Strain was calculated using the equation:

$$Circumferential\;Strain\;(\%)=\left(1/2\right)\;({\left(D_{sys}/D_{dia}\right)}^{2}–1)\times 100$$

Where D_sys_ represents the inner systolic aortic diameter, and D_dia_ represents the inner diastolic aortic diameter.

Inner aortic diameters were measured on the abdominal aorta at the PPE applied region of the aorta by ultrasonography in basic-mode-images at three different time points during a cardiac cycle. A morphological aortic size assessment of explanted aorta was performed of the aneurysmal abdominal aortic region. Outer aortic diameters were visually measured after laparotomy. Mean values were then calculated for each aorta. The dilation was calculated using the equation given below, Percentage change in diameter of aorta was calculated using the equation as a percentage change (%) = ((D2/Dd1)/D1) × 100 Where, D1 = initial aortic diameter, D2 = final aortic diameter

### Histology of aorta sections

Formalin-fixed 2–3 mm aorta pieces were processed in Tissue-TEK® tissue processor (Sakura Finetek USA, Inc., Torrance, CA) overnight and embedded in paraffin. Five-micrometer aorta sections were mounted on positively charged glass slides and were baked overnight to let the tissue attach to the slide and melt paraffin. Then the slides were deparaffinized with xylene following hydration with graded ethanol. Slides with aorta sections were stained for Verhoeff-van Gieson (VVG) (Richard-Allen Scientific, San Diego, CA) to visualize the elastin fibers. Overall tissue morphology was assessed by staining aorta sections with hematoxylin and eosin (H & E).

### Immunohistochemical analysis of aorta sections

Paraffin-embedded aorta sections mounted slides were subjected to heat-induced antigen epitope retrieval with citrate buffer (Thermo Scientific, MA). The slides were incubated overnight at 4°C with primary antibodies for anti—MMP-9 (Rabbit anti-Mouse) (Invitrogen, Carlsbad, CA), Rabbit anti-Mouse anti—MMP-2 (R & D Systems, Minneapolis, MN), Mouse anti- CD-68 (Novus, Cambridge, United Kingdom), anti-Mouse Mac-2 (Cedarlane, Burlington, Canada), and anti-mouse TGFβ1 (R & D Systems, Minneapolis, MN). The sections were incubated with relevant secondary antibodies (Goat anti- Mouse) (Invitrogen, Carlsbad, CA). IHC staining was completed with IHC kit (Enzo Life Sciences, NY). Slides were visualized by 3-Amino-9-ethylcarbazole (AEC) (Vector Laboratories, Burlingame, CA) chromogens followed by an appropriate counterstain.

### Flow cytometry

Mice were anaesthetized, PBS perfusion was performed from the right ventricle to drain the blood from the vascular system, and the aortas were harvested, cleaned and minced into 2- to 3-mm pieces. Aorta pieces were incubated in 1-mL digestion solution {with 1mg/ml porcine pancreatic elastase (Sigma-Aldrich); 0.2 mg/ml Collagenase (Sigma); and 0.2 mg/ml DNAse I (Roche) in DMEM media for one hour at room temperature. Excess PBS was added to stop the enzymatic reaction, and the tissue was minced and meshed on 70 μm filters. Immunofluorescence staining was performed as our previous methods [11]. Briefly, cells were incubated with Fc block solution (purified anti-mouse CD16/CD32, clone 2.4G2, BD Biosciences) for 15 min at room temperature to prevent non-specific binding. For CD68 cell surface markers, cells were incubated with the fluorescently conjugated antibody APC anti—CD68 (clone FA- 11) (BioLegend, San Diego, CA) in the dark for 30 min at 4°C. For intracellular cytokine TGFβ1 staining, cells were permeabilized with fix/permeabilisation buffer (eBiosciences, San Diego, CA) for 15 mins before antibody PE anti—TGFβ1 (clone TW7-2089) (BioLegend, San Diego, CA) staining. After the extensive washing of cells in FACS buffer, the cytometric acquisition was performed on an LSR II CytoFlex (Becton Dickinson). Data analysis was performed using FlowJo (TreeStar, Ashland, OR) software.

### Statistical analysis

Statistical analysis was conducted using GraphPad Prism 8 (GraphPad Software, San Diego, CA). Comparisons were performed by one-way or two-way ANOVA as appropriate. A posthoc test for multiple comparisons was performed. The significance was determined as p < 0.05. Logarithmic transformation was conducted before statistical analysis for percentage change for statistical analysis.

## Results

### Confirmation of nanoparticle targeting to the AAA

**Fig 2:**
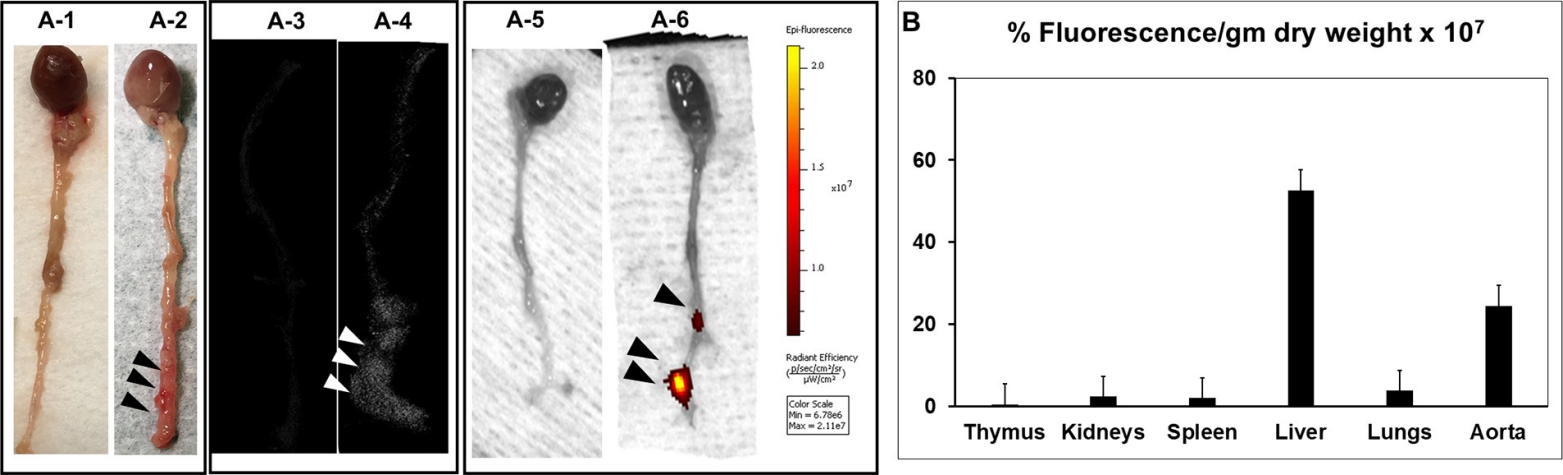
NP targeting to the aneurysmal aorta. **A-1 and A-2.** Representative picture of the explanted whole aorta from control and PPE treated mouse showing the aneurysmal region of the aorta. **A-3 and A-4**. Localization of EL-GNPs within aneurysmal tissues at Day-14 with attenuation model of micro CT. The signal of EL-GNPs is visibly stronger in the aneurysmal aorta which is localized with the expanded region. The control aorta is devoid of signal. **A-5 and A-6.** IVIS images of control and PPE treated mouse aortas. The fluorescence for EL-DiR-NPs is visibly strong in PPE applied regions of aorta showing targeting to degraded elastin, while the PBS applied aorta is devoid of fluorescence. **B.** Bar graph showing the biodistribution of EL-DiR-NPs quantified by the percentage of fluorescence per gram of dry tissue in different organs measured by IVIS imaging. The fluorescence was found significantly localized in the aneurysmal aorta confirming the targeting of nanoparticles.

**Fig 3:**
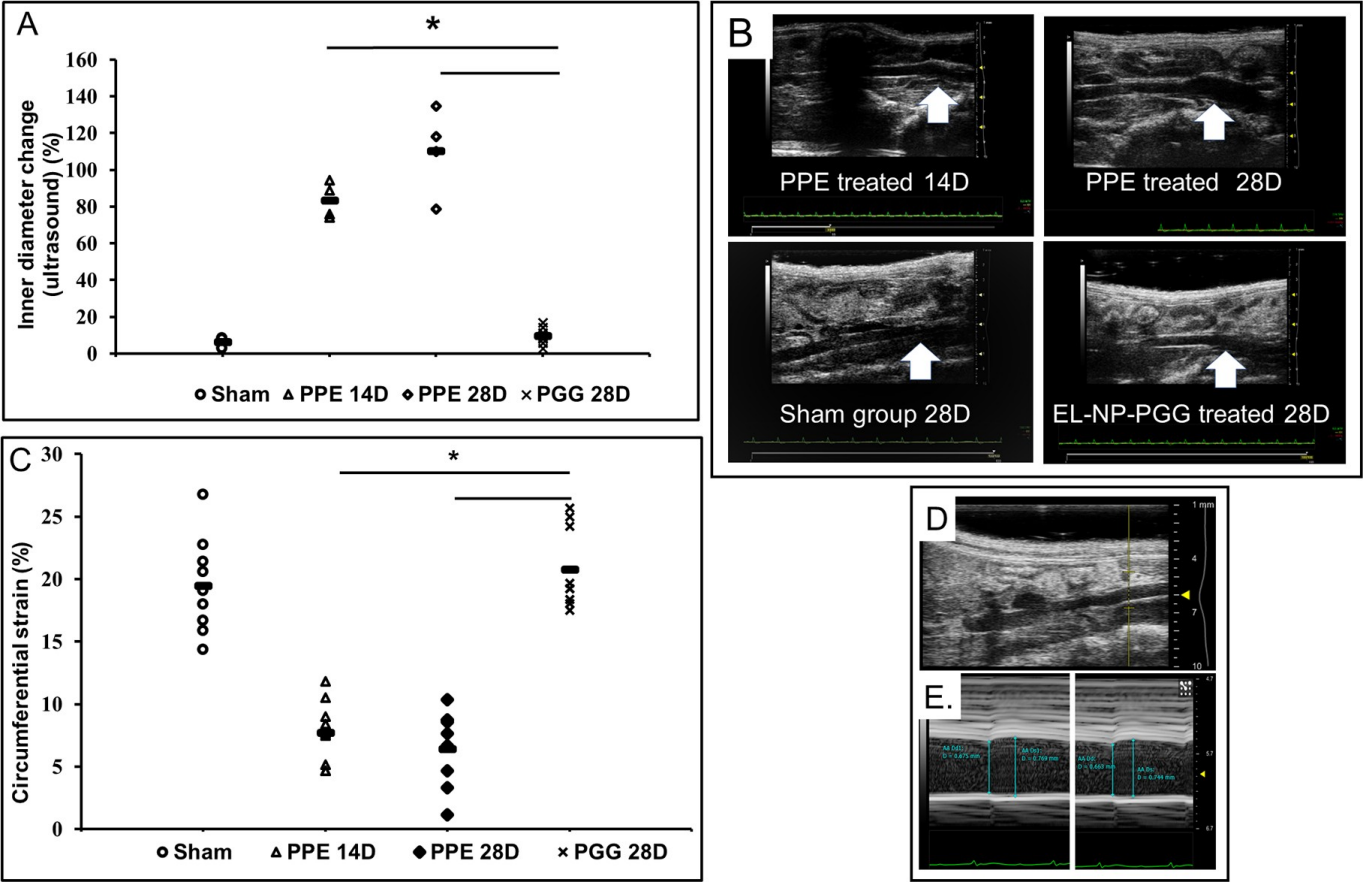
Reversal of aneurysm after PGG nanoparticle treatment. Representative data of the internal diameter of the aorta as measured by ultrasound. **A.** Representative scatter plot showing the internal diameter of the aneurysmal region of abdominal aorta decreases significantly after the PGG nanoparticle treatment (EL-PGG-NP) in comparison to elastase treated control (PPE) group. * p < 0.0001 as compared to control. **B.** A representative B-mode ultrasound *in vivo* scan of aortas showing the internal diameter decreases in EL-PGG-NP group. The size of aneurysm increased from Day-14 to Day-28 in the control group, and it was noticeably decreased after PGG nanoparticle treatment. **C.** Scatter plot of circumferential Green-LaGrange strains throughout the cardiac cycle. The control group showed decreased strain, suggesting stiffening of the aorta due to the loss of elastin. In the EL-PGG-NP group, the circumferential strain of aneurysmal aortas increased significantly aortas were similar to sham control at Day-28 (p < 0.0001) showing improved elasticity. **D and E.** Representative M-mode *in vivo* ultrasound images of abdominal aorta showing the measurement of systolic and diastolic diameter.

**Fig 4:**
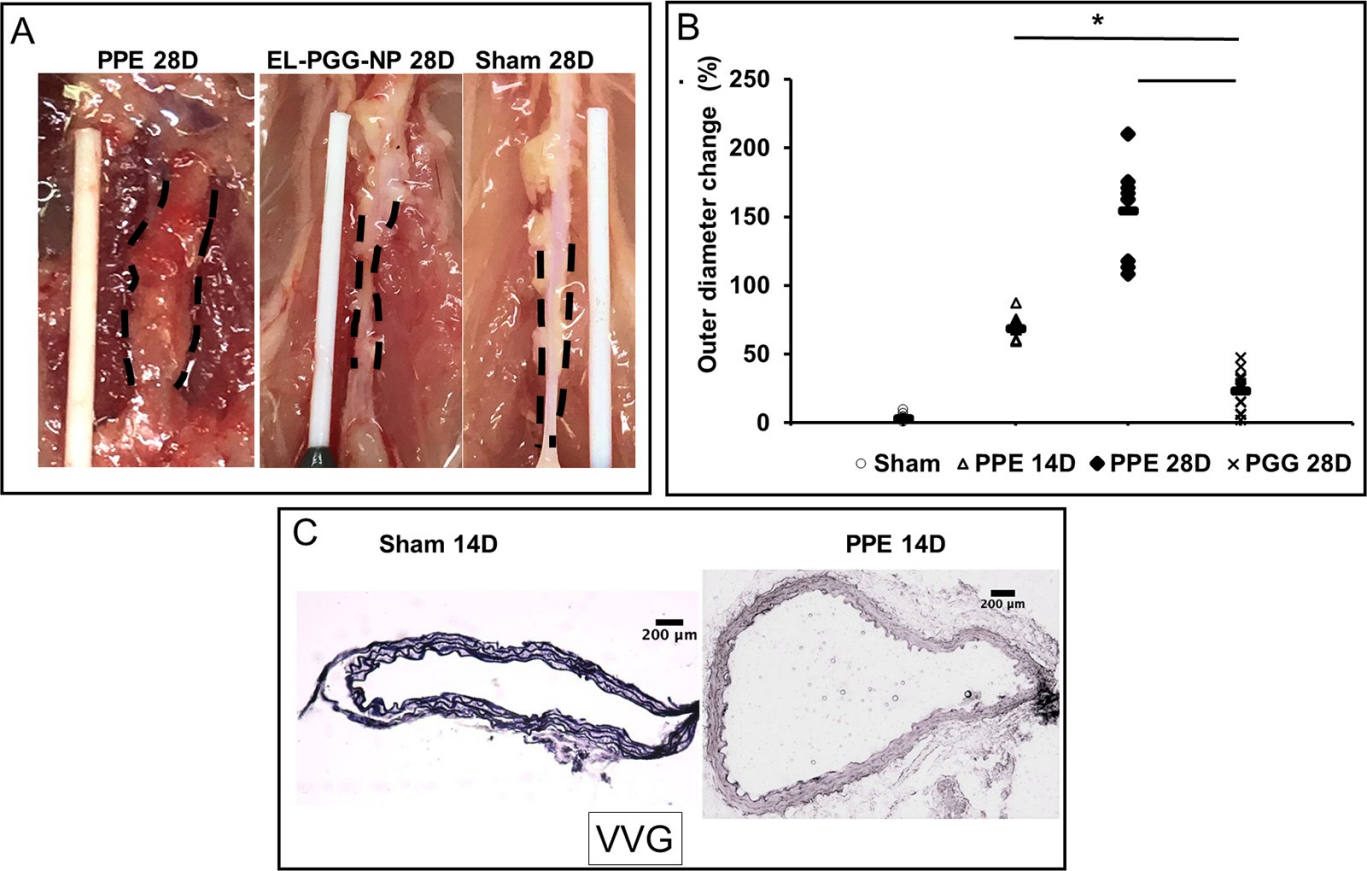
PGG nanoparticle treatment decreases the external diameter of aorta and adventitial inflammation. **A.** Representative gross pictures of aortas at 28 d. The aorta is visibly dilated in PPE treated mice at the abdominal aortic regions with a significant inflammatory capsule. The PGG nanoparticle treated mouse aortae (EL-PGG-NP) were not aneurysmal and looks similar to sham controls (n = 10 per group). **B.** The outer diameter of PPE treated aorta expanded up to 70% on day 14, and continue to expand to ~150% at day 28. After the two treatments of PGG nanoparticles starting at day 14, the external diameter of the aorta significantly decreased. * p < 0.0001 as compared to PPE treated controls. **C.** Representative histological sections (VVG stain for elastin- black) of PBS and PPE treated mouse aorta sections showing significant elastin damage at Day-14 at which point the PGG nanoparticle therapy was initiated.

**Fig 5:**
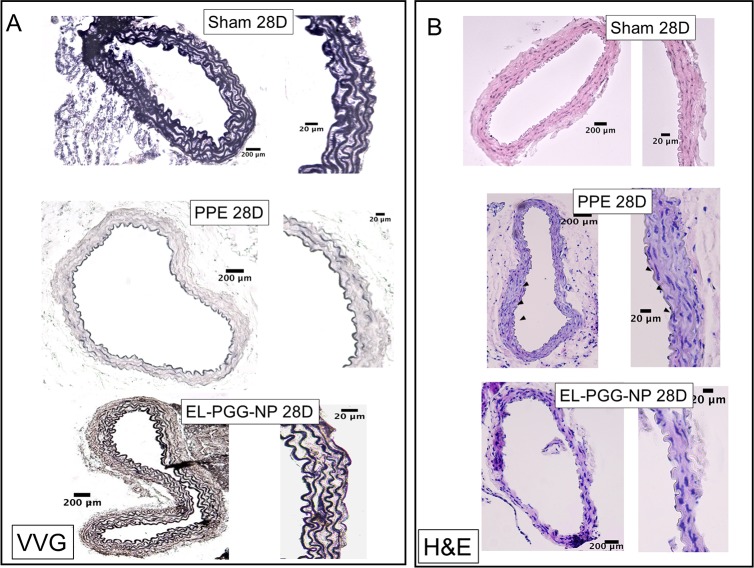
Restoration of elastic lamina after PGG nanoparticle therapy. **A.** VVG staining at 28d explants shows significant degradation of the elastic lamina in controls and regeneration of elastic lamina in the PGG treatment group (EL-PGG-NP). **B.** Hematoxylin and Eosin staining of PBS (Sham), PPE, and EL-PGG-NP group mouse aorta sections. Elastase treatment (PPE) caused significant adventitial inflammation while the EL-PGG-NP group showed normal aorta similar to sham.

**Fig 6:**
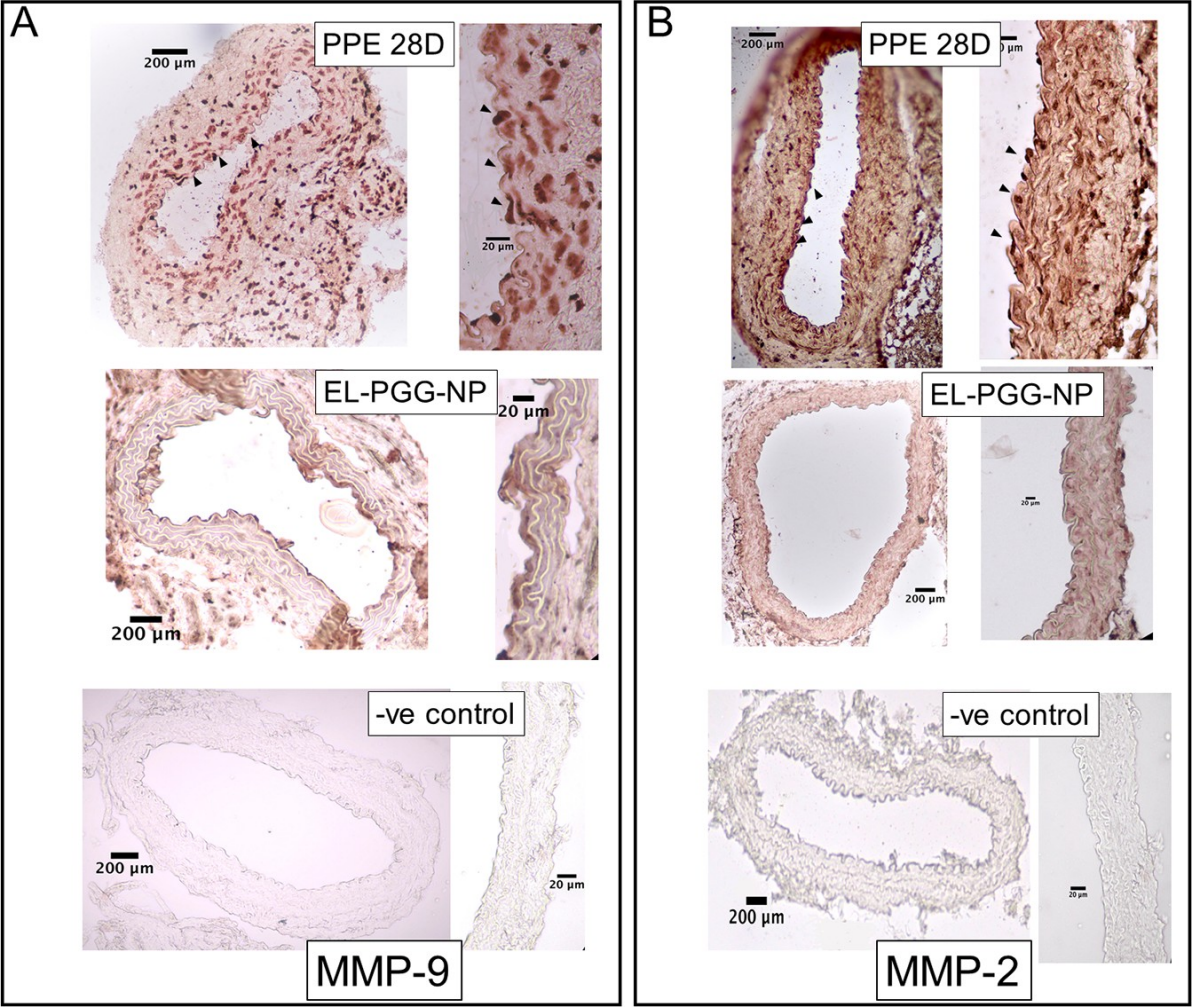
Reduction in MMP-2, MMP-9 and Mac-2 after the treatment of PGG nanoparticles. A. IHC staining of elastase treated (PPE), PGG nanoparticle treated (EL-PGG-NP), and PBS (Sham) treated mouse aorta sections, PGG treatment decreased MMP-9 staining in comparison to PPE treated aorta sections. **B.** PGG treatment also reduced the MMP-2 signal in contrast to PPE treated mouse section. **C.** IHC staining for Mac-2 macrophages in mouse aorta sections showing PGG treatment decreases the infiltration of macrophages in the medial layer of the aorta in comparison to that in PPE treated aorta.

To test if elastin antibody conjugated nanoparticles target AAA site, first, we confirmed the delivery of EL-GNPs (with micro-computed tomography, microCT) and EL-DiR-NPs (with IVIS imaging). AAA was allowed to develop for fourteen days after elastase treatment (Fig 2A-2). At day-14, EL-GNPs and EL-DiR-NPs were injected through the tail vein. Twenty-four hours after delivery, EL-GNPs were detected at the site of AAA with 3D reconstructed microCT image of the PPE treated aneurysmal aorta (Fig 2A-4) in the region of dilation of the aorta but no gold signal detected in sham control aorta (Fig 2A-3). Similar targeting of NPs to AAA site was found for EL-DiR-NP group with fluorescence located at the AAA site (Fig 2A-6). in comparison with the sham control (Fig 2A-5). Histology showed elastin damage area co-localized with the signal of DiR-NPs similar to our previous study [3]. We measured the biodistribution of EL-DiR-NPs in thymus, kidneys, spleen, liver, lungs and aorta. The AAA site showed highest fluorescence intensity per gram of tissue after liver, confirming antibody bound NPs were effectively delivered to the AAA site. A very little fluorescence per weight basis was seen in other organs (Fig 2B).

### PGG delivery regresses already developed aneurysms

The internal aortic diameter of PPE treated mice significantly increased above 75% at day-14, and it continued to increase up to 150% at day-28 (Fig 3A). At day 14, when AAA was developed, systemic delivery of EL-PGG-NP (once a week for two weeks) lead to a significant decrease in the internal diameter (below 20%) (Fig 3B).

The circumferential strain of the PPE treated aneurysmal aorta significantly decreased both at day 14^th^ and 28^th^ (Fig 3C), suggesting the loss of elasticity and stiffening of the aorta. After the intravenous delivery of EL-PGG-NP, the circumferential strain significantly increased in comparison to PPE treated group on day 28 and reached to sham control values (Fig 3C). The representative ultrasound images show how the circumferential strain was obtained at diastole (Fig 3D and 3E).

The gross pictures of the aorta after laparotomy at day-28 explant showed increased external diameter and significant inflammation in the PPE group (Fig 4A). The outer diameter of the aneurysmal aortas increased significantly above ~ 95% at day-14 and up to ~200% at day-28 in the PPE treated mice (Fig 4B). The histology at 14-day explant confirmed aneurysmal expansion and significantly degraded elastic lamina in PPE treated group (Fig 4C). While external diameter continued to increase in untreated group, in EL-PGG-NPs group, the external diameter significantly decreased below ~ 45% in comparison to PPE treated group at day-28 (Fig 4B). These data suggest that site-specific delivery of PGG with nanoparticles reverses already developed aneurysm and restores arterial mechanics [12–15].

### PGG delivery restores elastin and decreases inflammatory cell infiltration in the aorta

Histology with VVG stain showed significant degradation of elastin in the PPE treated aorta section compared with sham at both 14-day and 28-day explants (Fig 4C and Fig 5A). We initiated PGG nanoparticle therapy at day 14 when elastin was already fragmented (Fig 4C). After two weeks we found in the EL-PGG-NP group that elastic fibers were restored in the medial layer (Fig 5A). In H& E staining PPE treated aorta section was found thickened with infiltration of inflammatory cells in the aorta (Fig 5B). In EL-PGG-NP group, darkly pigmented nuclei of cells were sharply decreased, implicating the decrease in the infiltration of inflammatory cells (Fig 5B).

### PGG delivery decreases matrix metalloproteinase (MMPs) in the aorta

MMPs degrade ECM, specifically elastin, and play a vital role in the development of AAA [16,17]. These MMPs are secreted by inflammatory cells that are infiltrated in the aneurysmal aorta [18]. A significant amount of MMP-9 and MMP-2 on IHC staining were observed on PPE treated aorta sections both in adventitia and media of the aorta. Those levels were significantly decreased in the EL-PGG-NP treatment group (Fig 6A and 6B). These results indicate that the systemic delivery of EL-NP-PGG for two weeks decreases matrix metalloproteinases (MMPs) especially MMP-9 and MMP-2 activity.

### PGG treatment decreases macrophage cells in the aorta

**Fig 7:**
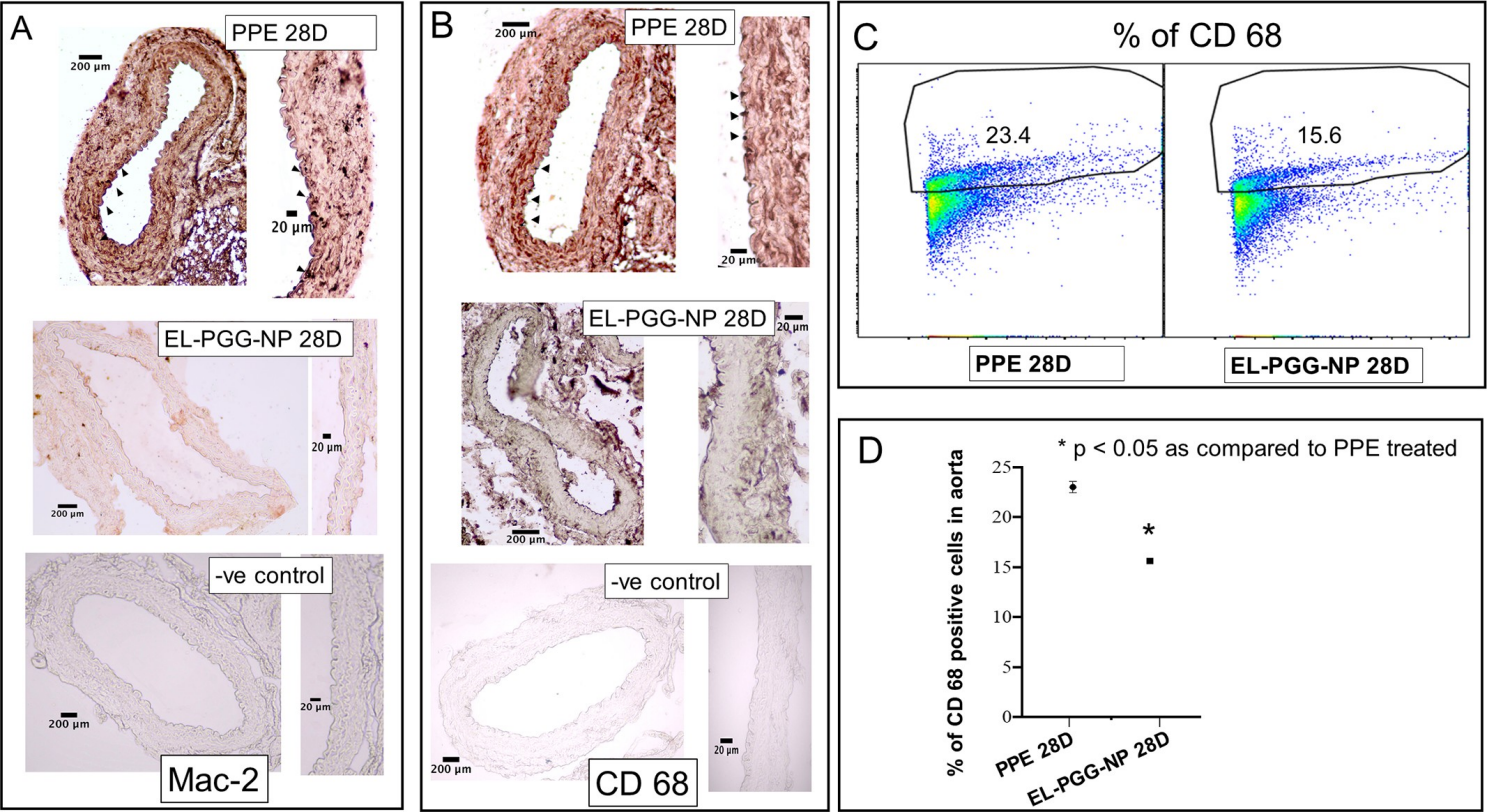
Reduction of Mac-2 and CD 68 macrophages in the aorta after the PGG nanoparticle treatment. **A.** IHC staining of PPE, PGG (EL-PGG-NP), and PBS treated mouse aorta section showing PGG treatment decreases the infiltration of Mac-2 macrophages. **B.** IHC staining of PPE, PGG (EL-PGG-NP), and PBS treated mouse aorta section showing PGG treatment decreases the infiltration of CD 68 macrophage in the medial layer of aorta section. **C.** Flow cytometry scatter plot showing after PGG treatment decreases CD 68 macrophage in PGG treated aorta region. **D.** A scatter plot showing the percentage of CD 68 macrophages decreases significantly in PGG treated aorta region in comparison to that in PPE treated aorta. * p < 0.0001.

Immunofluorescence staining was conducted for CD 68 cells, which is also known as pan-macrophage and is regarded as an M1 macrophage marker [19]. We observed that after the treatment with PGG, CD 68 positive cells significantly decreased in the aneurysmal aorta in comparison to PPE treated aorta (Fig 7B). Similarly, CD 68 positive cells were significantly decreased in aneurysmal aorta measured by immunofluorescence staining (Fig 7C and 7D). Moreover, a massive number of activated macrophages of Mac-2 positive cells observed in the PPE treated aortic sections, while the Mac-2 positive cells significantly decreased in the EL-PGG-NP group (Fig 7A), especially in medial layer. These results suggest that the PGG treatment reduces the macrophage cells in the aorta that may be due to the decrease in overall inflammation in the aorta.

### PGG treatment decreases TGFβ1 in the aneurysmal aorta

**Fig 8:**
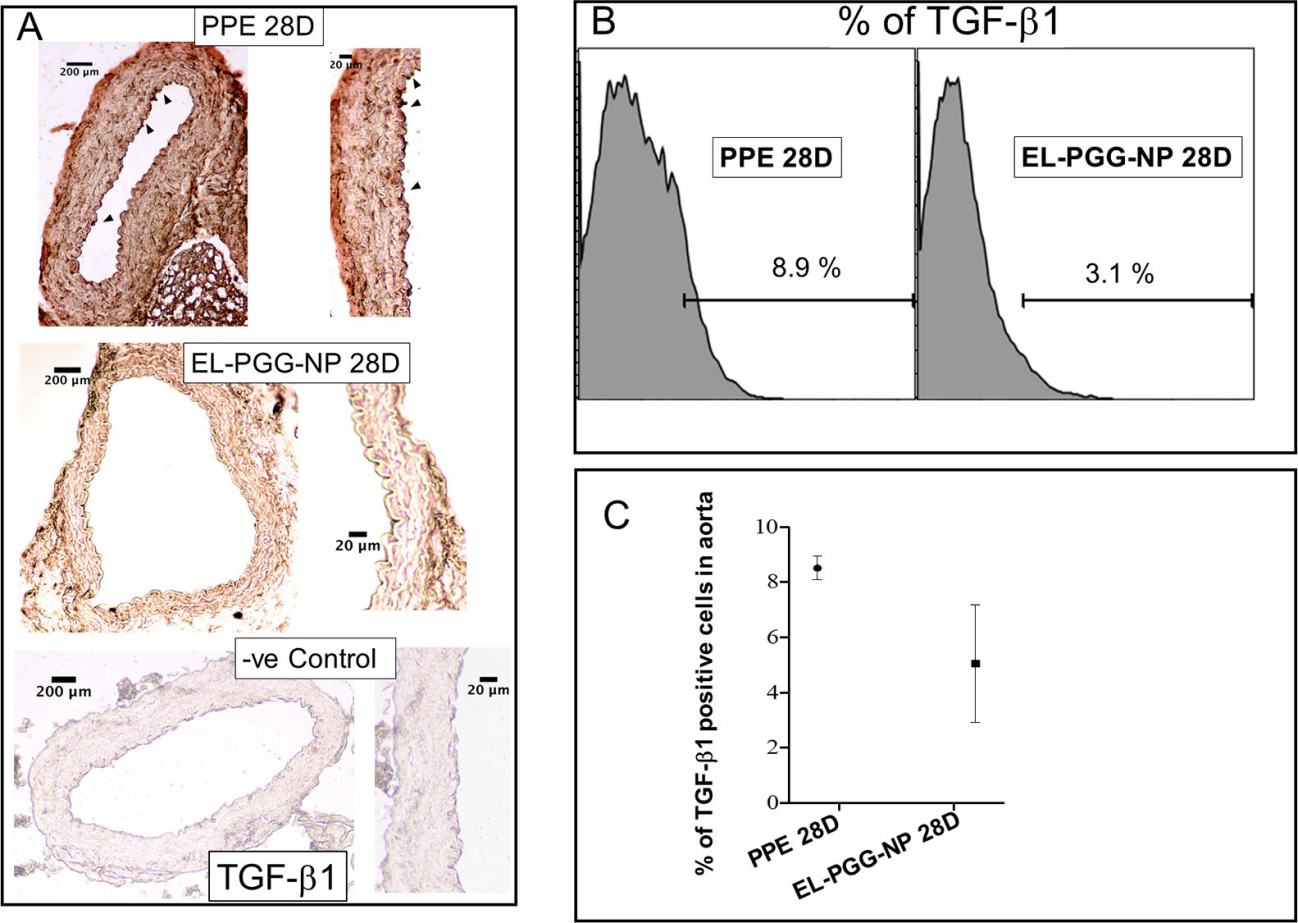
A reduction of TGF-β1 after PGG nanoparticle treatment. A. IHC staining of aorta section showing PGG treatment drastically decreased the staining of TGF-β1 in aorta section in comparison to PPE treated aorta. **B.** Flow cytometry histogram and **C.** Scatter plot showing PGG treatment decreases the percentage of TGF-β1 in comparison to that in PPE treated aneurysmal aorta.

TGFβ1 plays a crucial role in the progression of aneurysm. Others have shown that TGFβ1 administration exacerbates aneurysms [20,9]. IHC staining for TGFβ1 was performed to assess the presence of TGFβ1 in the aneurysmal aorta, in PPE and PGG treated aorta sections. We observed that TGFβ1 was heavily present in the PPE treated aneurysmal aorta (Fig 8A), while after the treatment with PGG nanoparticles (EL-PGG-NP group), flow cytometry showed a decrease in intracellular TGFβ1 in the aneurysmal aorta in comparison to PPE treated aorta (Fig 8B).

## Discussion

The purpose of this study is to test if our novel targeted NP based PGG delivery that only targets degraded elastin could reverse already developed aneurysms in elastase-induced AAA. We not only show that such targeted delivery regresses aneurysms, but we also show that such therapy restores elastin lamina, decreases matrix metalloproteinase (MMPs), immunomodulating cytokine TGFβ1, and infiltration of activated macrophages at the aneurysmal site.

Elastin is found in the major blood vessels of nearly all vertebrates formed of a pulsatile, high-pressure closed circulatory system [21]. Elastic fiber imparts reversible distensibility to the large arteries, allowing the aorta to deform during cyclic hemodynamic loading, with no permanent deformation or energy dissipation upon load retrieval [22]. One of the significant consequences of aneurysm development is the degradation of the elastic lamina. Unfortunately, adults cells cannot regenerate lost elastic fibers on their own as the microfibrillar assembly is lacking [23]. Furthermore, degraded elastic lamina can release elastin degradation products (EDPs) that are chemokines for inflammatory cells and modulate M1/M2 macrophage polarization [24]. Thus, stopping elastin degradation is a crucial step in preventing AAA development. We previously reported local PGG treatment of stabilized arterial elastin and prevented degradation of elastin [12]. We have also shown that PGG treatment of vascular smooth muscle cells allowed the development of crosslinked elastin in cell cultures [4]. In the calcium chloride rat AAA model, dual-targeted therapy reversed calcification and aneurysmal expansion [3].

Here we chose a more aggressive model of AAA where the local application of elastase significantly degraded elastin in the medial layers by day-14. We applied porcine pancreatic elastase (PPE) for 12 mins, which developed variable dilation after two weeks and showed significant elastic lamina degradation in all mice (Fig 4C). Bhamidipati et al. used PPE for 10 mins, and with an oral dose of doxycycline showed prevention of the progression of AAA [25]. Similarly, Wang et al. applied PPE for 30 mins and used an oral dose of grape seed polyphenol to show prevention of AAA development. However, the amount of grape seed polyphenol given daily (400–800 mg/kg) is excessive (correspond to 28–55 gm dose per day in humans)[10].

Similarly, Setozaki et al. showed green tea polyphenol Epigallocatechin-3-gallate (ECGC) given in drinking water prevented AAA formation and reduced elastin degradation and inflammation in a combination of elastase and CaCl_2_ mediated AAA in rats. They also used excessive amounts (correspond to 60-80L green tea consumption as it has low ECGC content). Furthermore, most previous studies initiated therapies at the onset of elastase treatment to prevent AAA formation and did not target drugs to the site of the aneurysm. We wanted to find out if targeted therapy could reverse already developed AAA in this aggressive model. Such targeted treatment would need a small amount of drug released at the site to reverse aneurysms.

We used our novel nanoparticles coated with elastin antibody that only targets degraded elastin while sparing healthy elastin to deliver PGG to the AAA site [3, 6, 13, 26]. We chose albumin-based NPs as they are proven to be non-toxic [27] and are used to deliver paclitaxel in patients (Abraxane). We previously demonstrated that EL-PGG-NPs have no hepatic toxicity for systemic delivery, and these particles are targeted at the aneurysmal site [3].

Here we clearly show that nanoparticle reach and adhere to the AAA site in the elastase model. Both albumin-based loaded with DiR dye and gold nanoparticles could be targeted at the AAA site by conjugating nanoparticles with an antibody that only recognizes degraded/fragmented elastin. Twenty four hours after nanoparticle delivery we found significant nanoparticles at the AAA site. A substantial portion of the nanoparticles was also seen in the liver, which is very common to nano/microparticle delivery as the liver is the processing organ [13,28]. We have shown earlier that nanoparticles are retained at AAA site while they clear out from the liver within two weeks [3]. Once we confirmed that site-specific delivery to AAA site was possible, next, we delivered PGG loaded nanoparticles on day-14 to test if PGG delivery could restore elastin that is already degraded by elastase treatment and reverse AAA. It is important to point out that we started our therapy on day 14 after elastase treatment where substantial elastin fragmentation and aneurysm development was already taken place (Fig 4C). We performed two systemic injections of nanoparticles one week apart as we have shown that PGG slowly releases from particles over a period of several weeks, and nanoparticles stay at the site for more than a week [13]. Histology after two weeks of PGG delivery (28 days after elastase treatment) showed elastic laminae in the medial layers were restored in the aneurysmal aorta in PGG treated mice (n = 10), while elastin degradation further continued in control group. Such restoration of elastic lamina also led to a significant reduction in the aneurysm size shown by both internal and external aortic diameter measurements. We also demonstrated that PGG significantly increased aortic circumferential strain. Circumferential strain can provide some insights for in situ biomechanics of the aorta. As AAA progresses, aorta becomes stiffer due to the loss of elastin and elastic recoil. Thus, recovering of the circumferential strain after PGG treatment to similar levels of sham group demonstrates improved in the biomechanics of the aorta. The previous report shows aneurysmal aorta’s circumferential strain is inversely correlated with the elastin fiber loss and directly associated with the diameter decrease [29]. Thus, not only PGG treatment restored elastic lamina and reversed AAA but allowed aorta to restore properties to that of the healthy aorta.

### PGG mechanisms of action in reversing AAA

During the progression of AAA, accumulation, and activation of inflammatory cells takes place in the aorta. These inflammatory cells play a key and substantial role in vascular remodeling [9, 30]. In our study, we found a significant amount of CD 68 (M1 type macrophage) and activated macrophage Mac-2 in PPE treated aorta. Macrophages secrete a wide range of inflammatory mediators such as IL-13, IL-6, and TNFα, and overexpression of these inflammatory mediators is reported in experimental AAA followed by the infiltration of monocytes, neutrophils and lymphocytes and then followed by VSMCs apoptosis [19,4]. These inflammatory cells and apoptotic VSMCs release MMPs, which degrade ECM leading to vessel damage [31]. Macrophages produce profibrotic mediators such as TGFβ1 and control the homeostasis of various matrix metalloproteinases and tissue inhibitors of matrix metalloproteinases [32,33]. In our study, after the treatment of PGG nanoparticles, CD 68 and activated macrophages Mac-2 drastically decreased in the aorta. The reduction of macrophages might have contributed to the reduction in inflammation and, thus, for the amelioration of aneurysm. We have reported it before that PGG binds strongly to elastin and prevents its further degradation by elastases [12]. Such binding can avoid the generation of elastin peptides that are known chemoattractant to macrophages [34]. Thus, we hypothesize that PGG binding to elastin stops this inflammation, MMP secretion, and ECM degradation loop that exacerbate AAA progression.

In this study, we observed MMP-9 and MMP-2 decreased after the treatment of PGG nanoparticles. MMP-9 and MMP-2 are required for the development of AAA since MMP-9 and MMP-2 knockout mice are resistant to AAA disease [35]. Others have shown that polyphenols like curcumin and xanthohumol inhibit matrix metalloproteinase activity and even have anti-inflammatory effects [36, 37]. The MMP activity reduction with PGG could have occurred in two ways. First, it could directly inhibit MMPs as shows by us that PGG inhibits MMP activity in vitro by gel zymography [38]. It is also possible that the prevention of macrophage recruitment is due to inhibition of elastin fragmentation by bound PGG to elastin, as shown above, further decrease local MMP activity.

In this elastase model, we not only saw a reduction in inflammation, MMP activity and reversal of aneurysmal dilation but also the restoration of lost elastic fibers in the aorta. Previously we have reported that polyphenols PGG and EGCG regenerate elastin in vitro in VSMC and pulmonary fibroblasts cell culture and in vivo in CaCl_2_ model of AAA. PGG interacts with monomeric tropoelastin to enhance coacervation, then PGG activates vascular SMCs to increase LOX production thus enhancing the crosslinking of coacervated elastin[3,4]. Therefore, it is possible that the local presence of PGG allowed anchoring of tropoelastin secreted by cells for further crosslinking by LOX at the site of AAA.

Immunomodulatory cytokine TGFβ1 plays a key role in ECM modulation. TGFβ1 neutralization either worsened or mitigated aneurysm formation, depending on whether treatment was initiated before or after the establishment of the aneurysm in a thoracic aorta [20]. TGFβ1 contributed to the progression of the aneurysm when administered after the formation of the aneurysm in the experimental mouse model [9,20,39]. Depending on circumstances, TGFβ1 signaling may follow the Smad2 dependent or independent pathway for its effect on the pathogenesis of aneurysm formation. Healthy elastic fibers are known to sequester TGFβ1 through latent TGFβ binding protein (LTPBs) that is associated with microfibrils of elastic fiber [40]. Thus, a healthy extracellular matrix modulates TGFβ activity, and loss of LTPBs due to degradation of elastin can increase the activity of TGFβ1. In this research, we observed that significant levels of TGFβ1 and Mac 2 were present in the PPE treated aneurysmal aorta. We found that after the treatment of PGG nanoparticles, TGF β1 was significantly decreased in the aorta. That might be due to the reduction of monocyte and macrophage infiltration in the aorta. Research has shown that monocytes and M2 phenotype macrophages secrete TGF β1 [41,32]. It is possible that the restoration of the healthy matrix in the aorta led to a decrease in TGFβ1 activity in the aorta. These data suggest that PGG may further decrease the pathogenicity in aneurysm by decreasing TGFβ1 signaling in AAA [9,39]. It is noteworthy that a minimal amount of PGG was required for the reversal of AAA. We used 10 mg/kg nanoparticle dose, and PGG loading in nanoparticle was 30%. Thus, only 3 mg PGG was used two times in two weeks with an adequate dose of ~425 μg/day/kg for two weeks; therefore, a small locally targeted PGG can be effective in reversing AAA.

In conclusion, in our study, site-specific delivery of PGG with our targeted nanoparticles that accumulate in the AAA site reversed already formed aneurysms, regenerated elastic lamina, reduced macrophages, and inflammatory cytokines and MMPs in the aorta. The therapy also restored the healthy mechanics of the aorta. Such treatment can be translated to patients who already have AAA disease as there are no therapies available to reverse AAA.

## Supporting information

S1 File Supplementary methods- preparation of gold nanoparticles, methods for microCT and ultrasound data. (DOCX)

S1 Fig Size distribution of different batches of nanoparticles. **A.** Data showing size distribution of G-NPs. **B.** Data showing size distribution of EL-DiR-NPs. **C.** Data showing size distribution of EL-PGG-NPs.
(TIF)

S2 Fig Scatter plot showing local pulse wave velocity (PWV) of the aorta (meter per second) in the mouse. Representative scatter plot of Pulse Wave Velocity of PBS treated (Sham), elastase treated (PPE 14D), elastase treated (PPE 28D), and PGG nanoparticle treated (PGG 28D) mouse aorta.
(TIF)

## Abbreviations

AAA: Abdominal aortic aneurysm
BSA: Bovine serum albumin
DIR: 1,1-Dioctadecyl-3,3,3,3- tetramethylindotricarbocyanine iodide
EL-GNP: elastin antibody conjugated gold nanoparticle
EL-NP: Elastin antibody-conjugated blank nanoparticles
EL-PGG-NP: Elastin antibody-conjugated and PGG-loaded albumin NPs
FACS: Fluorescence-activated cell sorting
MMP: Matrix metalloproteinase
NPs: Nanoparticles
PGG: Pentagalloyl glucose
TGF-β: Tumor Growth Factor beta
IL: Interleukin
