# Supplementary material for: Nanoparticle-based targeted delivery of pentagalloyl glucose reverses elastase-induced abdominal aortic aneurysm and restores aorta to the healthy state in mice
Source: PLoS One. 2020 Mar 27;15(3):e0227165. doi: 10.1371/journal.pone.0227165 (PMC7100957; doi:10.1371/journal.pone.0227165)
Supplement: S1 File — (DOCX) [file pone.0227165.s001.docx]

**Synthesis of Gold Nanoparticles**

Gold GNPs were synthesized with the procedure described by Bastus et al. 2011 ^1^ ^2^. Briefly, 25 mM solution of gold III chloride trihydrate (HAuCl4) (Sigma - Aldrich, St. Louis, MO) was slowly injected into a continuously heated (at 90^0^C) 250 ml (2.2mM) sodium citrate buffer filled vessel supplemented with a condenser to control evaporation. The mixture was vigorously stirred for 15 mins at which the particle color changed from yellow to bluish gray and then to pink (after 10 mins). These GNPs obtained are caped with negatively charged citrate ions and their particle size was found ~ 10 nm. For analysis 55 ml of sample was extracted, and subsequently added 54mL of sodium citrate buffer and 1 mL of HAuCl4 separately. To get larger particles size, the process was repeated several times by stirring the solution under continues heating as in previous cycle for 10 in the same reaction condition. Each repeated step increased the particle size of GNPS by ~ 10nm. The process was repeated until the GNPs size reach up to ~ 150 nm size distribution (S­­_Fig 1A).

**S­_Figure 1:Size distribution of different batches of nanoparticles. A.** Data showing size distribution of G-NPs. **B.** Data showing size distribution of EL-DiR-NPs. **C.** Data showing size distribution of EL-PGG-NPs.

**Gold nanoparticles (EL-GNPs) conjugated with anti-elastin antibody preparation**

Gold nanoparticles (GNPs) obtained with an average size of ~ 150±25nm were PEGylated with a heterobifunctional thiol-PEG-acid (SH-PEG-COOH) (2000MW, Nanocs, New York, NY) was added at a ratio of 4:1 by weight for PEGylation and was stirred at 4°C for 48 hrs to achieve PEGylation. PEGylated GNPs were washed in MES buffer by centrifugation. Anti-elastin antibody was conjugated with PEGylated GNPs by adding EDC/NHS according to the manufacturer’s protocol ^2^. Briefly, EDC (N-(3-Dimethylaminopropyl)-N′-ethyl carbodiimide hydrochloride) (Oakwood Chemical, Estill, SC) and Sulfo-NHS (N-hydroxysulfosuccinimide) (Sigma Aldrich, St. Louis, MO) were added at a ratio of 2:1 and 4:1 (by weight) separately to the PEGylated GNPs and incubated for 6 hours and collected after centrifugation. Anti-elastin antibody (custom-made at Clemson University) at the rate of 4µg per mg GNPs were incubated overnight at 4 °C. The anti-elastin conjugated GNPs (EL-GNPs) were delivered by intravenous injection.

**Micro-computed tomography (Micro-CT) scanning of the aorta**

After 24 hrs. of intravenous delivery of EL-GNPs, mice (n=6) were sacrificed and excess PBS was perfused to flush out the blood, aorta was cleaned and immersed in corn oil, and then scanned (90kV, 250mAs, 300ms, 0.2mm Al filter) with a Skyscan 1176 high-performance micro-CT system (Bruker, Billerica, MA). The Skyscan NRecon software using the Feldkamp algorithm was used to reconstruct the images. The reconstructed images were visualized using DataViewer and CT-Vox software. The intensity of the signal of EL-GNPs within the aortas was determined by using 3D attenuation images.

**Calculation of Pulse Wave Velocity (PWV)**

Ultrasound imaging (Vevo2100, VisualSonics, Toronto, Canada) was performed in mice kept in a supine position on the imaging table with 1-3% of isoflurane inhalation. The aortic stiffness parameter pulse wave velocity was calculated from the images in the M-Mode and EKV modes by using the following formulae.

PWV= Distance / Transient time (T_2_-T_1_)

Where Distance = the length of the edges of the box

Transient time= is the difference between the time starting from the foot of the peak of ECG peak to the downward and upward feet of the pulse waves respectively ^3^.

**PGG Treatment decreased local Pulse Wave Velocity (PWV) of the aorta**

Pulse wave velocity which is the marker of stiffness was found increased in PPE treated aortas (both at 14D and 28D) and found to be decreased in El-PGG-NP treated group (Figure S_Fig 2). This observation suggests that PGG treatment increased the elasticity of aorta that might be the result of PGG’s contribution to the restoration of vascular wall including elastin.

**S_Figure 2. Scatter plot showing local pulse wave velocity (PWV) of the aorta (meter per second) in the mouse.**

Representative scatter plot of Pulse Wave Velocity of PBS treated (Sham), elastase treated (PPE 14D), elastase treated (PPE 28D), and PGG nanoparticle treated (PGG 28D) mouse aorta.

**References**

1. Bastus NG, Comenge J, Puntes V. Kinetically Controlled Seeded Growth Synthesis of Citrate-Stabilized Gold Nanoparticles of up to 200 nm: Size Focusing versus Ostwald Ripening. *Langmuir* 2011;**27**:11098-11105.

2. Wang XY, Lane BA, Eberth JF, Lessner SM, Vyavahare NR. Gold nanoparticles that target degraded elastin improve imaging and rupture prediction in an AngII mediated mouse model of abdominal aortic aneurysm. *Theranostics* 2019;**9**:4156-4167.

3. Karamched SR, Nosoudi N, Moreland HE, Chowdhury A, Vyavahare NR. Site-specific chelation therapy with EDTA-loaded albumin nanoparticles reverses arterial calcification in a rat model of chronic kidney disease. *Sci Rep* 2019;**9**:2629.
