# Supplementary figures and images for: Nanoparticle-based targeted delivery of pentagalloyl glucose reverses elastase-induced abdominal aortic aneurysm and restores aorta to the healthy state in mice
Source: PLoS One. 2020 Mar 27;15(3):e0227165. doi: 10.1371/journal.pone.0227165 (PMC7100957; doi:10.1371/journal.pone.0227165)

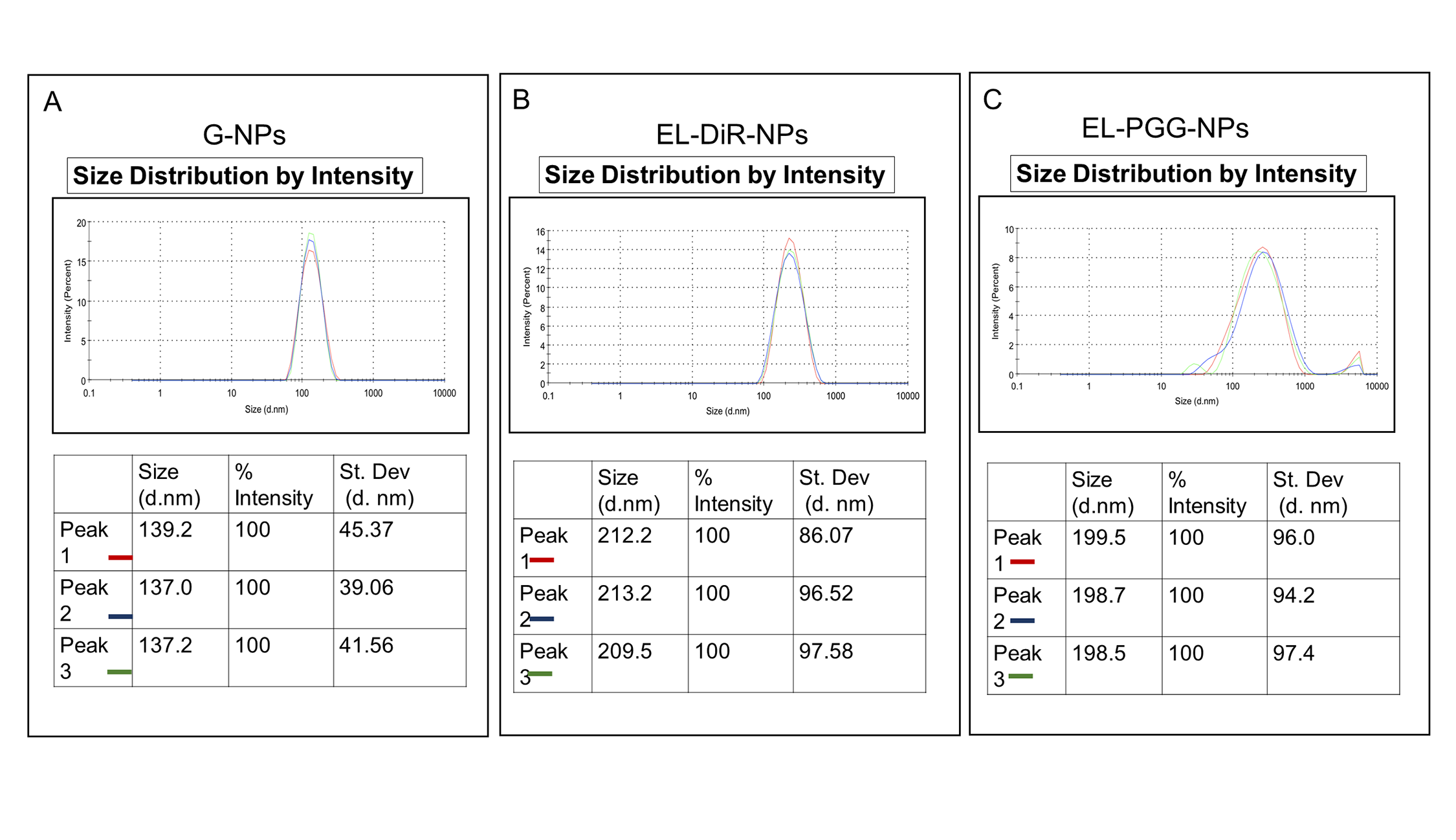

Supplement: S1 Fig — A. Data showing size distribution of G-NPs. B. Data showing size distribution of EL-DiR-NPs. C. Data showing size distribution of EL-PGG-NPs. (TIF) [file pone.0227165.s002.tif]

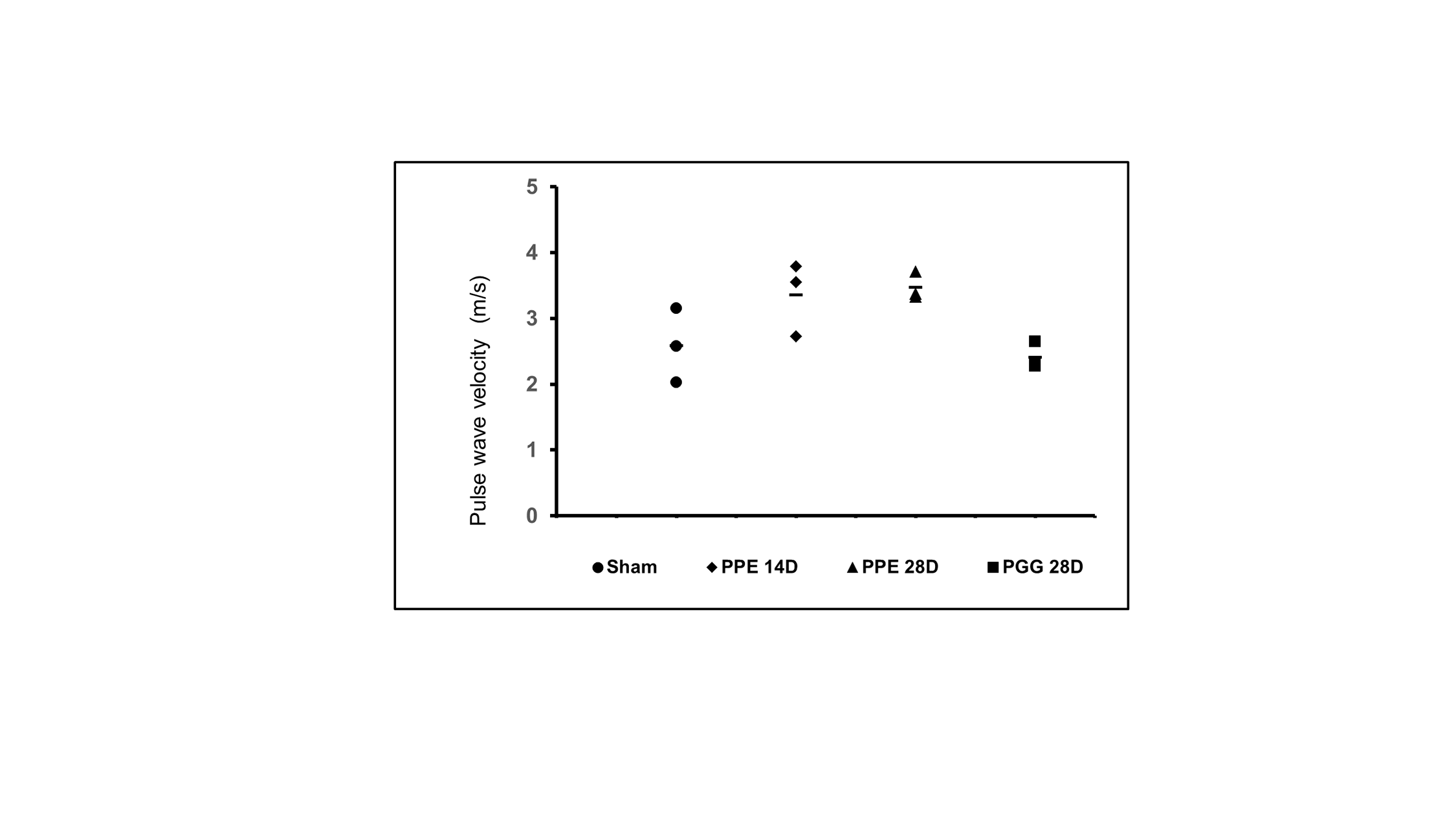

Supplement: S2 Fig — Representative scatter plot of Pulse Wave Velocity of PBS treated (Sham), elastase treated (PPE 14D), elastase treated (PPE 28D), and PGG nanoparticle treated (PGG 28D) mouse aorta. (TIF) [file pone.0227165.s003.tif]
